# Designing Place-Based Interventions for Sustainability and Replicability: The Case of GO! Austin/VAMOS! Austin

**DOI:** 10.3389/fpubh.2018.00088

**Published:** 2018-03-22

**Authors:** Aliya Hussaini, Carmen Llanes Pulido, Semonti Basu, Nalini Ranjit

**Affiliations:** ^1^US Health, Michael and Susan Dell FoundationAustin, TX, United States; ^2^GO! Austin/VAMOS! Austin (GAVA), Austin, TX, United States; ^3^Measurement and Evaluation, Michael and Susan Dell Foundation, Austin, TX, United States; ^4^University of Texas Health Science Center, Austin, TX, United States

**Keywords:** place-based interventions, social-ecological model, coalition-building, community change, sustainable interventions, obesity, built environment

## Abstract

Place-based health efforts account for the role of the community environment in shaping decisions and circumstances that affect population well-being. Such efforts, rooted as they are in the theory that health is socially determined, mobilize resources for health promotion that are not typically used, and offer a more informed and robust way of promoting health outcomes within a community. Common criticisms of place-based work include the difficulty of *replication*, since engagement is so specific to a place, and limited *sustainability* of the work, in the absence of continued institutional structures, both within the community and supporting structures outside the community, to keep these initiatives resilient. This paper describes a place-based initiative, GO! Austin/VAMOS! Austin (GAVA), which was designed to harness the strengths of place-based work—namely, its specificity to place and community. From the start, the project was designed to balance this specificity with a focus on developing and utilizing a standardized set of evidence-informed implementation and evaluation approaches and tools that were flexible enough to be modified for specific settings. This was accompanied by an emphasis on leadership and capacity building within resident leaders, which provided for informed intervention and demand building capacity, but also for longevity as partners, philanthropic, and otherwise, moved in and out of the work.

## Background

GO! Austin/VAMOS! Austin (GAVA) is a place-based, cross-sector initiative to improve the health of residents living in South Austin through improved access to healthy food and safe physical activity. GAVA was launched in 2012 by the Michael & Susan Dell Foundation in partnership with community organizations, schools, and residents. Implementation efforts began in the South Austin zip code of 78744 (Dove Springs) and expanded to the neighboring zip code of 78745 2 years later.

Although Austin is considered one of the healthiest cities in America, zip codes 78744 and 78745 face systemic issues that pose barriers to healthy living. They are the most populous zip codes in the city, but lack basic amenities. Parks in both zip codes lack basic features such as playscapes, water fountains, park benches, and have gone years without sufficient lighting to keep the neighborhoods safe. Fifty percent of adults in the community and 80% of the children in area elementary schools are overweight or obese (1). In 2012, a collective of nonprofit and governmental agencies came together to address the health needs of these communities, through the place-based GAVA initiative. Informed by social-ecological theory (2), GAVA holds that health is strongly influenced by the built environment and that organized communities are key to creating and sustaining demand for healthy opportunities and social norms supportive of healthy behaviors (3–5). GAVA seeks to both harness and improve existing built and social environments through a defined set of change strategies, *viz*.:

• Increase access to built environment assets, such as parks and healthy food, and couple these with demand building strategies, in order to influence healthy behavior changes (6, 7).
• Facilitate residents’ capacity to lead these efforts to increase relevancy, community ownership, and community use of such infrastructure (8–10).

GO! Austin/VAMOS! Austin was designed as a model for sustainable community change that could be translated to other communities in need. The sustainability strategy focused on coalition building (11, 12), both within the community and across implementing organizations. Thus, an important precondition was that communities had an existing reservoir of social capital that was necessary to facilitate and disseminate change strategies. GAVA is not merely community-placed; it is explicitly community-based, prioritizing the building of social capital reserves over making changes to the built environment (5). In addition, while the work in 78744 and 78745 was specific to the assets and needs in those individual communities, and responsive to the emergent and evolving needs of those who live, work, learn, and worship in the neighborhoods, the overall design of the GAVA project was informed by theory and created according to identifiable strategies that allowed it to maintain sufficient fidelity for evidence-informed strategies to be impactful and replicable in other urban communities. We hypothesized that over a 5-year period, this theory-driven, community-based intervention simultaneously targeting both built environment assets and resident capacity to utilize them would lead to measurable changes at the individual level (changes in diet and activity behaviors as well as access and utilization of community assets) as well as the community level (increases in number and quality of built environment assets).

## Building Sustainability

### Selecting Communities With Social and Financial Resources to Support Intervention

In identifying potential sites for the GAVA partnership, the aim from the beginning was to capture information that would not just illustrate the needs of the neighborhood, but also its assets (13). Need was defined as a combination of socioeconomic characteristics of residents, lack of environmental assets promoting healthy eating and physical activity, and childhood obesity prevalence rates. Given the place-based approach, it made sense to curate these numbers with a geographic focus. To that end, GIS maps of zip-code level demographics and of community infrastructure for food access, physical activity, and transportation were constructed. These were supplemented with Fitnessgram data collected in schools to identify obesity hotspots across the city (1). Together, this compilation of data presented a comprehensive picture of which zip codes had the highest need relative to available infrastructure and access points.

An equally important precondition for selecting the community was the presence of assets (physical, organizational, and human) that would be critical in *supporting* the intervention to address these needs (5, 14). To assess community assets, an inventory of community-based organizations that were located in or served each specific zip code was undertaken, focusing on those with strong leadership, those emphasizing partnership with their clients, and those with healthy, diversified funding streams. Community residents were engaged to determine their priorities and willingness to work on raised issues, and to begin to build relationships. Ground truthing through visits to the neighborhood with resident guides allowed us to validate the community relevance of data that we had obtained from the needs assessment, for example, whether green spaces were actually viable physical activity destinations.

Funding coming into the zip code from other sources was identified as a priority asset that would help sustain intervention activity. It was clear that larger, more durable infrastructure investments requiring an array of public partnerships and funding were outside the capacity of GAVA to deliver as part of the initial intervention. The City of Austin was queried to determine priority sites for infrastructure projects, and what they had observed with regard to community characteristics that would maximize potential success, such as community cohesion, and leaders who were able to successfully engage with the city.

This neighborhood analysis resulted in the foundation narrowing the focus to two specific zip codes to invest in. With a population of about 43,000, the 78744 zip code had the highest obesity prevalence at close to 30% among middle school children [the national average is 17% (15)] and also had the highest percent of the population under 18 years of the communities investigated (50%). Predominantly Hispanic, the median income was among the lowest, at $38,000, and more than half of the population were renters (16). Based on previous GIS studies, the community had very limited infrastructure with respect to healthy food and physical activity access. However, the zip code had a long history of community organizing to mitigate safety concerns, and a number of established neighborhood watch teams. They already had relationships with the City of Austin, including the Parks & Recreation Department and City Council, based primarily on these issues. Given their work to bring resources and increased public safety efforts to their neighborhood, they had established community leaders, a sense of community cohesion, and an established community agenda.

The analysis also revealed significant need and capacity in the neighboring community of 78745, but with a few notable differences from 78744. With a population of about 55,000, 78745 had a slightly higher median income than 78744 ($43K versus $38K) and fewer children on free and reduced lunch (82 versus 92%). The community also had a slightly lower obesity prevalence among students (26 versus 29% of middle school students) (1). What was lacking in 78745 was the community cohesion and leadership that was the hallmark of 78744. The community was larger in its geography, with a wide range of demographics (specifically regarding income and ethnicity), fewer long-term residents with more renters, and an upwardly mobile community that seemed to pass through more than established roots. Building community cohesion and identifying resident leaders was identified as the most promising engagement model for this neighborhood.

However, differences across these communities were of smaller magnitude than the commonalities. Both communities had significantly higher poverty and obesity prevalence than the national average. Significant mobility had been documented in both these communities. Choosing two, especially geographically contiguous communities across Austin’s I-35 corridor, created the opportunity for zip code-based efforts and messaging to reinforce each other, with the recognition that a mix of engagement models that made the most of foundation staff and funding capacity would be needed.

### Building Community Trust

Trust was a vital component to launching the work and remains a critical component to continuing efforts. Particularly, in Dove Springs, the community had been selected for a wide array of previous assessments and interventions. Residents were used to being in studies, asked their opinions, and then left to wonder what happened as researchers wrote a thesis or a needs assessment and moved on. This made many key stakeholders wary of a new partnership.

The foundation was, from the beginning, transparent about what was in scope of the initiative and what wasn’t. Dove Springs had a number of community priorities beyond those considered in scope. These included health-care access, affordable housing, economic development, and others. While there was tremendous interest in hearing all the barriers to wellness, only some would be addressed directly, and residents would need to be connected to partners for others. The foundation was prepared to listen and to show evidence that residents were being heard. When resident leaders were approached with a plan for engagement following the neighborhood analysis, they signaled dissatisfaction with the organizations selected to lead the work, primarily because these organizations were not located in the neighborhood and did not have an established relationship with the residents. A 1-h meeting scheduled to share the implementation plan turned into a 4-h meeting to devise a new one. The foundation continued this strategy—responding to resident input with changes that reflected feedback, even if it meant significant changes in the way the foundation did work internally to accommodate what residents thought would work in the field, and built in mutual accountability for results.

Efforts were also directed toward building trust with the entities that were already known and familiar to the community. Knowing that it would take significant time to build trust within the neighborhood, particularly given the long history of failed relationships with interventionists, evaluators, academicians, and others, the foundation worked to gain the trust of trusted entities. It was not an easy path, but it was a clear one, with non-negotiables and a strong desire to approach the work as equals.

### Building a Leadership Structure Within the Community

Because GAVA is a coalition-based initiative and not an independent 501(c)3 organization, there is no board of directors. The leadership structure of the initiative has been created to allow the space for resident leadership and organizational partnership and guidance. It consists of the following core components:

• Coalition—a group of community decision makers who represent the various sectors, community based and service organizations, and residents and leaders of the community. Coalition meetings are attended by institutional partners, funded implementation partners, local policy makers, and most importantly, residents in the community. The agenda of the coalition meetings is centered around providing updates of progress made on GAVA goals using data and maps, identifying priority areas of work based on resident feedback, and providing for capacity building opportunities such as leadership training.
• Advisory Council—to guide the coalition’s decision-making and maintain accountability to the partner communities, the GAVA coalition created an Advisory Council, comprised of director-level representatives from partnership organizations, and leaders from the resident-stakeholder teams. A simple majority of residents and community stakeholders is maintained on the Advisory Council; votes are taken to endorse various decisions that affect the coalition.
• Executive Director—the director functions as the key strategist to lead the work within the neighborhoods (17). This key leader, with an organizing background, has a strong history with neighborhood residents and a deep understanding of community challenges and opportunities.

## Structured Implementation and Evaluation Models

The specificity and thus the limited generalizability, of place-based interventions has generally been accepted as given in the field of health promotion (18). Yet, it is evident that the learning and insights from any given intervention should have lessons for other interventions, to maximize the impact of population health promotion efforts. Replicability of GAVA’s implementation model was seen as a key objective in structuring the intervention. Elements of replicability were built into both the implementation and evaluation tools and activities, so that GAVA could offer a model for other place-based interventions. In the past 2 years, we have seen considerable interest from other funders, government and nonprofit entities in learning how GAVA has built up the coalition and successfully engaged residents in the place-based initiative. As a result, the Executive Director has developed a training manual to offer guidance and tools for replication in other communities, and the implementation team is developing a series of training offerings to provide technical assistance and share best practices. Some of the key insights from implementation and evaluation are documented below.

### Utilization of a Sectoral Approach

One of the first decisions regarding the organization of GAVA activities was that it would follow a sector-based framework for delivery (19, 20). Five sectors of activity were identified consistent with the goals of the overall project: physical activity access through assets like parks and greenspaces, healthy food access through farm stands and corner stores, early childhood, coordinated school health, and neighborhood safety addressing pedestrian safety and crime challenges. Selection of these sectors, informed by the focus on children and their families, and the goal of meeting them where they were was facilitated by community leaders. Of these five sectors, schools were assigned a central role. Schools have always been a central hub for foundation obesity prevention efforts, but data from district wide implementations of the Coordinated Approach to Childhood Health (21) and Healthy Schools Program demonstrated the importance of an engaged surrounding community, especially parents, and organized coordinated school health teams. GAVA’s goal was to effectively activate the power of parent advocacy, which had proven elusive in many past implementations, and to integrate coordinated school health efforts into a more activated community, working toward similar goals. Elementary schools in the community were selected based on feeder pattern to the middle school in the neighborhood, and relationships were established with principals and parent leaders to get buy-in from the start of the intervention. The single middle school in the zip code was included for continuity of messaging and strategies as families moved across the educational spectrum. In Dove Springs, one of our implementing partners and a core trusted neighborhood resource was located at the zip code’s middle school. Some of our other strategies, including those focused on food access and access to physical activity, utilized the locational advantages of schools (although they were not limited to schools). Thus, farm stands were set up at schools, joint-use agreements were set up with school playgrounds, and parks in schools were among early physical activity facilities targeted for improvements.

The sectoral implementation strategy, while guided by local needs and the availability of local resources to address these sectors, is easy to adapt to other settings. It has the advantages of being relatively modular, so that the implementation strategy mix within each sector is complimentary and coordinated with but not entirely dependent upon the activities in other sectors. Further, it leverages different community strengths and funding resources as and when these become available (22).

### Utilization of Evidence-Based Strategies

A menu or master list of evidence-based strategies and tools was developed to identify the implementation strategies to be implemented by GAVA teams in each of the sectors. This menu was initially developed by conducting an extensive literature search for evidence-based strategies for each sector (13), and informed by the foundation’s previous investments in the space. The menu was further informed by stated community preferences, as well as by anticipated availability of funds for particular implementation strategies. Over time, the menu has evolved to include a finite set of “gold standard strategies” organized by site type (school, park/green space, food retail environment, early childhood care site, etc.) that are research-based, as well as applicable to the community. The use of the term “gold standards” is idiosyncratic to GAVA and was utilized to emphasize to community residents that ultimately, all strategies that they chose to implement would need to be connected in some way to the master list. Because the strategies were sourced from both the literature and community members, the evidence-basis for these strategies was not always available or defined. Nevertheless, all strategies were scored by a team of implementation and University-based evaluation experts according to the following rubric. Scores were developed for Effectiveness (1–3) based on literature review; Reach (1–2) based on the anticipated numbers of people directly impacted within the community setting; and frequency (1–2) depending on the length of time for which the strategy is implemented. The product of these three scores gives a total Impact rating, or “dose.” The impact score was calculated for each strategy. The entire gold-standard document of strategies, with impact rating for each, forms the basis for the suite of strategies any given site-based team is undertaking. Further details describing the identification of strategies and development of a usable menu are available online (23). While dosage is monitored to encourage implementation of highest impact strategies, action planning is based on community input, with implementation driven by resident and neighborhood team leaders whose lived experience provides the context and approach for strategies to be more specific and effective. The rubric we created is an elaboration of the “dose of interventions” rubric advocated by Kaiser Permanente (24).

### Leveraging Geographic Teams for Sector Activities

Organizing the implementation through teams in each sector kept the funding mechanisms and initial workplans streamlined, because we had subject matter experts in the form of community-based nonprofit partners in each sector. However, when 78745 became active, one of these partners, an affordable housing nonprofit, provided an opportunity to engage resident teams in multi-sector efforts that affected their own family and immediate community (25). This new model of micro-neighborhood or geographic (geo) teams was replicated in other small parts of the zip code and encompassed relevant access points for that area in each of the sectors. Two geo team organizing positions were created within GAVA’s headquarters, and strong, cross-sector teams began to develop within other local housing complexes and around schools, parks, and stores. This strategy of geo team organizing was so effective that after 2 years of engagement with 78745, the neighborhood had achieved the same level of community readiness as had been developed in many years of organizing in 78744.

### Structured Ongoing Evaluation

A key goal of evaluating strategies was that results from evaluation be available and transparent to residents and stakeholders at all times, so as to usefully inform the direction of their implementation efforts. This required then that evaluation be an ongoing activity, rather than a one-time exercise at the end of the intervention. This applies to impact evaluation, but even more to process evaluation (26), which contains more actionable information in most cases.

#### Process Evaluation

Because the process evaluation directly guides implementation efforts, investment was made in collecting and reporting activities and key outputs to implementation teams on an ongoing basis. There are two key sources of process data available to inform implementation. Site-based teams produce site plans at the beginning of each year, or at the start of a new team. Updated quarterly, these plans are based on the gold standard list of strategies described earlier, i.e., comprised of evidence based and evidence informed strategies curated for each type of site—schools, early childhood education sites, parks and greenspace, food retail outlets, and so on. In addition, “Key wins” data are collected from teams monthly and organized by categories under the three themes of *sustainability, access, and utilization* (Table 1). Examples of key wins include leveraged funding, policy progress, resident leadership development, building of organizational networks, and implementation wins like infrastructure improvements, safety improvements, creation of new healthy food access points, and health education/outreach.

**Table 1:** Implementation outcome categories targeted by GAVA.

Sustainability	Access	Utilization
Development of community teams and leaders who drive wins rooted in evidence-based strategies	Infrastructural improvements that support physical activity	Physical activity and nutritional programming and education

Policy and funding wins	Improved community safety, i.e., traffic calming, crime prevention	Engagement in healthy behaviors and healthy modeling

Creation of institutional networks and partnerships	Increased healthy food sources Increased awareness of new community assets	Increased physical activity (PA) and fruit/vegetable consumption (FVC)

**Figure 1:**
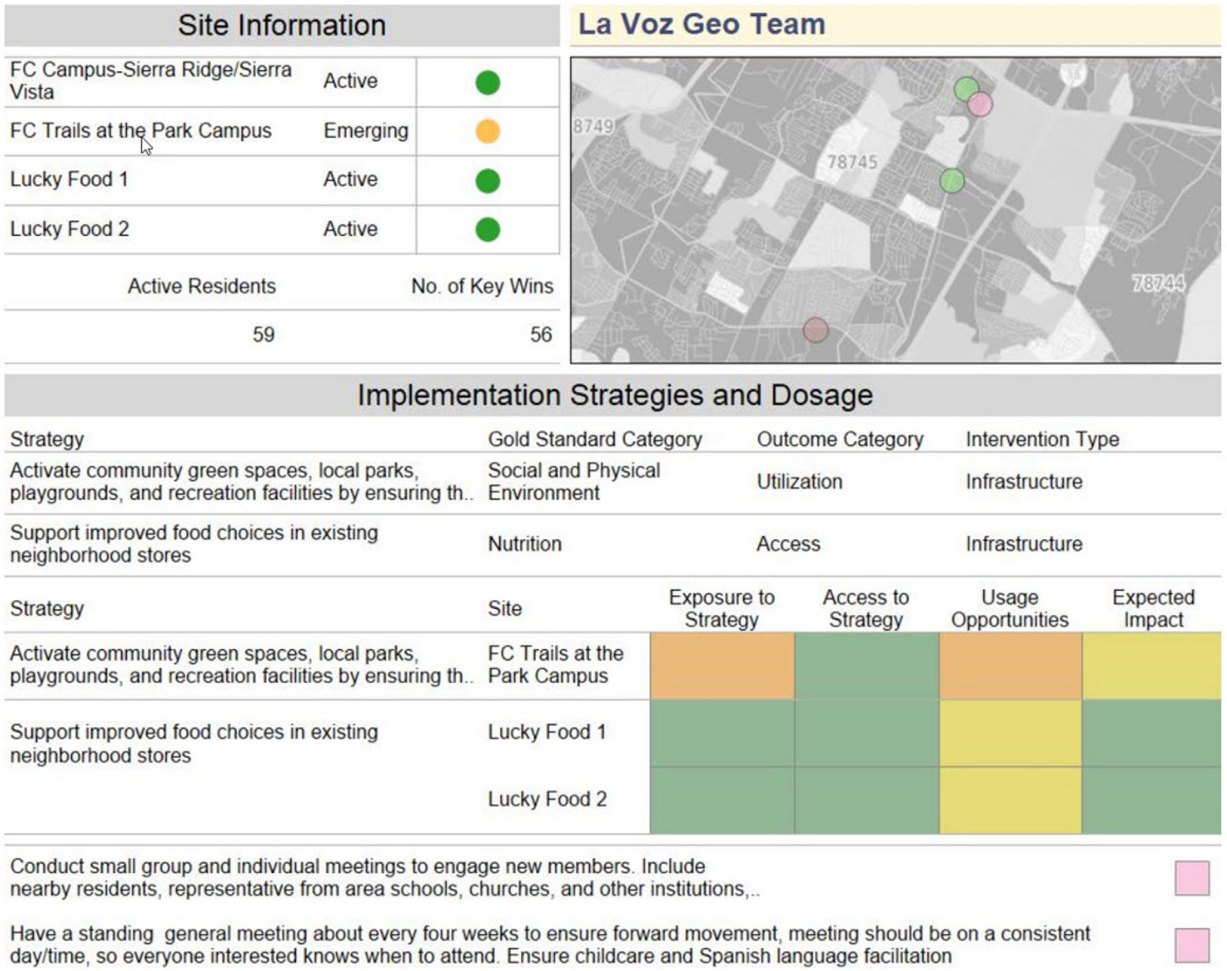
Example of site-specific dashboard data presented at monthly meetings.

Key wins and site plan data are summarized in a monthly dashboard (see Figure 1) to provide insights into implementation progress as well as to ensure strategic alignment across all levels of GAVA stakeholders. This allowed the dashboards to be presented at meetings comprised of the Executive Director, the funders, the impact evaluation team, the communications team, and core GAVA personnel. Findings from the dashboard were often used to discuss implementation priorities with the funder, as well as discuss gaps that may need attention from the operations staff. Highlights from the dashboard, as well the highlights of the discussion were then summarized and presented periodically at operations staff meetings to ensure alignment among implementation team. Key wins data were also shared with community residents in the form of maps, to generate interest among neighborhood residents on GAVA priorities. Site plans and key wins data served as progress reporting for the funder as well as the broader GAVA coalition. The monthly implementation dashboards were also used by GAVA’s Executive Director to display active versus emerging teams with their geolocation; current active implementation strategies along with their impact score; categorization of the type of intervention (infrastructure, policy, and so on) and expected outcome category (increased access, increased site utilization, and so on). The dashboard also displays the number of active individuals and accumulating key wins for the teams. The dashboard view (as shown in the accompanying figure) allowed the Community Director and the GAVA implementation teams to monitor activity of a multi-component intervention with clarity.

*Map visualizations* of process and impact data have been essential to the coalition, enabling partners to see what types of barriers residents are identifying around particular sites, what those same residents might be looking for with regard to improved access, and more recently, where utilization rates of healthy access points have increased. An example of the kind of map visualization that is used is presented in Figure 1. Being able to see results at the street level helps the implementation to maintain its micro neighborhood focus, allowing for the nuances of what can make place-based efforts so successful. However, the structure of this visibility, evidence-based gold standards, and outcomes analysis, provide the standard structure that can make place-based efforts more replicable. For example, residents around one corner store reported that availability of healthy fruits and vegetables was a barrier to access for healthy food, while residents around another corner store reported that fruits and vegetables were available but too costly or of poor quality. Implementation plans for the teams focused on these two retail outlets were variable, despite their proximity.

#### Impact Evaluation

The impact evaluation for GAVA is being conducted independently by an evaluation team at the University of Texas Health Science Center at Houston School of Public Health, Austin Campus. As is the case with GAVA implementation activities, the evaluation plan was designed to be comprehensive and flexible to accommodate changing ground realities, but also follow the structure of the implementation. As a result, the evaluation plan consists of several sub-studies and uses mixed methodology. The cohort sub-study is a 5-year longitudinal study in which the research team follows 150 families living in the GAVA community and 150 families living in socioeconomically similar communities outside the GAVA community. Respondents report their awareness, attitudes, perceptions, and behaviors in regard to use of physical activity and healthy eating opportunities provided in their community, as well as physical activity and dietary behaviors of an index child. In addition, the evaluation team tracks objectively measured BMI data from study participants and from the index child over the 5-year period. Data from the cohort study will allow us to determine the impact of GAVA on child and parent outcomes by comparing intervention versus control families. The cohort study is supplemented by a serial cross-sectional survey, the door-to-door (D2D) study that collects data from approximately 300 individuals living in the GAVA communities each year. The D2D participants self-report many of the same constructs as the cohort participants, but is limited to adult data. Addresses of both cohort and D2D participants are obtained and geocoded, to allow examination of place-based intervention effects. In addition to these two quantitative studies, the evaluation team conducts yearly interviews with school principals (the principal interview sub-study) and with key stakeholders and community residents (the community readiness sub-study). Finally, the evaluation team conducts observations of community assets to determine changes in quality of these assets over the evaluation period.

**Figure 2:**
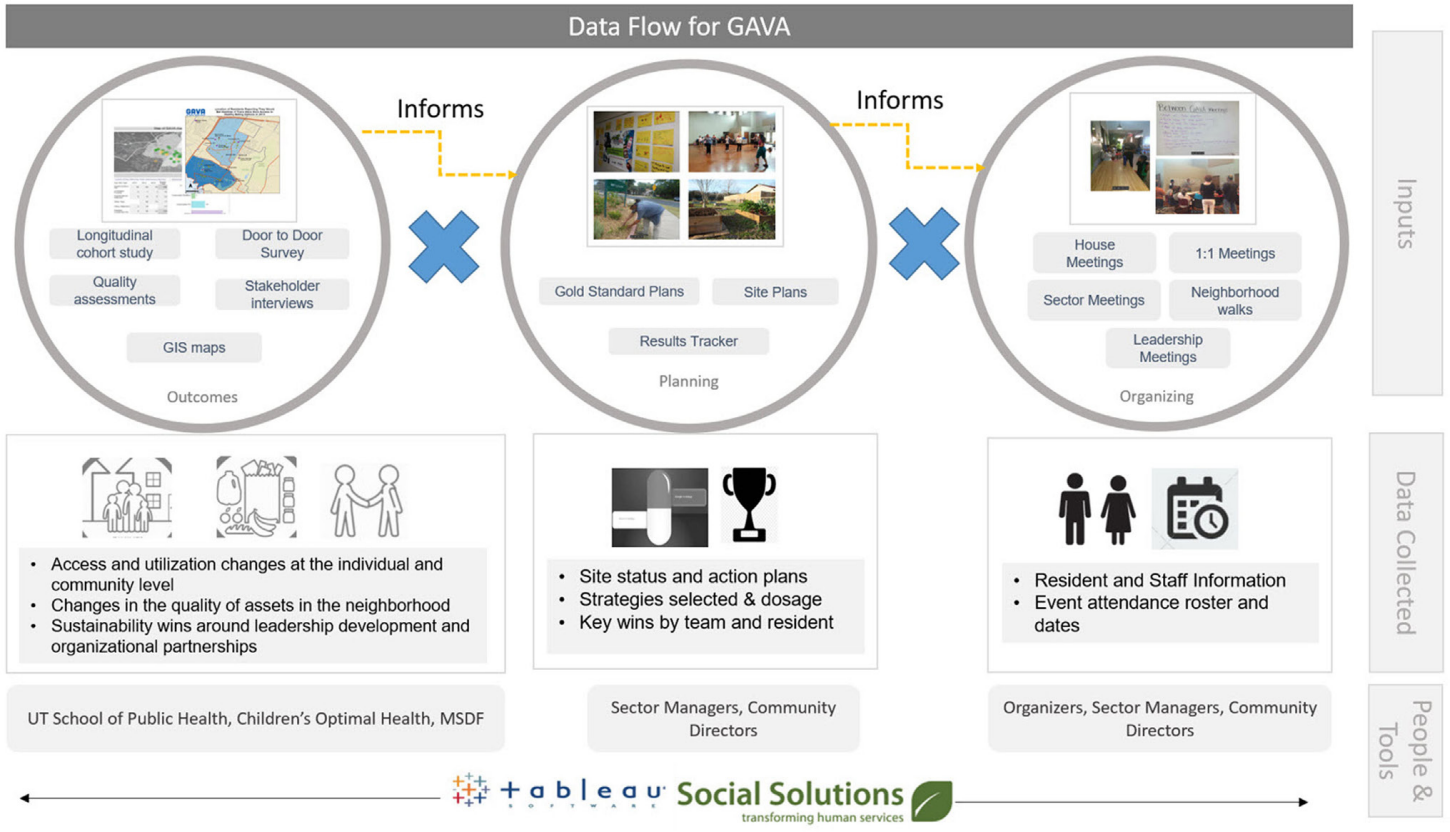
Flow of evaluation and process data within GAVA.

Data from the cohort and D2D surveys are compiled annually and summarized as feedback for implementation team, both to allow monitoring of progress, as well as to provide information that serves to redirect resources to areas of high need or low impact, identify specific actions to strengthen fidelity of implementation and address access and quality gaps with regard to healthy food and physical activity. Figure 2 shows how interim evaluation data feeds into process data. These interim data summaries from the cohort and D2D teams have also proven useful to the GAVA leaders as advocacy tools and in pursuing funding to maintain viability and sustainability of GAVA.

### GAVA Outcomes at the Community and Organizational level

GO! Austin/VAMOS! Austin is now in year 5 of a planned five-year implementation in the first zip code, and year 4 of implementation in our second. At this time, the GAVA coalition is comprised of six funded non-profit/city agencies, over 122 organizations, and over a thousand residents. Of the 98,420 individuals living in both zip codes, 86% (84,641 individuals) have geographic access (living within a one-mile radius) to GAVA assets. Over 1,600 residents have been mobilized as part of GAVA outreach efforts, and over 800 residents are active leaders in GAVA implementation across both zip codes. Currently, 35 implementation teams comprised of residents, community leaders, and institutional staff are focused on park and creek adoption, coordinated school health, early childhood, and increased access to healthy foods and local produce. Over the course of 3 years, resident led teams made improvements to more than 32 community assets *via* key wins around infrastructure improvements, policy, and funding changes and programming (numbers as of March, 2016).

In areas with high residential leadership, key wins include a 30% reduction in perceptions of barriers to physical activity. Reduction in access barriers are geographically correlated with increased usage of the neighborhood assets, and improvements in physical activity utilization behaviors by 20%. Similarly, mobilization of resident activity around awareness building and outreach is correlated with a 20% increased awareness of barriers to healthy food access. Positive trends in nutrition have also been observed with increased utilization of local food retail outlets.

Last year, the foundation funded the work at 50% of operational costs, this year at 33%, and next year at 33% in only the second neighborhood launched. This trajectory has required identification of supportive funding from private and public sources for operational activities. Additionally, organizations that participate in GAVA and see the increased value in having engaged client advocates have pledged to support key staff who benefit their organization and GAVA overall, through current or raised operational funds. The GAVA implementation team is now spinning off independently as an organization to best maintain and deepen core operational functions while serving as partners to other institutions and initiatives in the execution of community agendas for population health.

## Conclusion

Given the way GAVA is organized, the tools used to monitor process and gaps, and the measurement that has been put in place, there are strong indications as to what structures and strategies are working and can be replicated. The building of partnerships and networks far outstretch the geography of the current implementing neighborhoods and further support an effort aimed at replication with fidelity. We are also considering a broader set of social determinants to begin addressing in more established GAVA neighborhoods with the capacity and will to do so.

The GAVA team has often been asked whether funding resident leadership, or capacity building within people, is actually sustainable. Indeed, GAVA makes the case that the investment in people is the key to sustaining these efforts. GAVA communities are among the most mobile of Austin residents—GAVA works because it does not depend on a small cadre of leaders. Rather, capacity building within residents, and progression through a pipeline of leadership, has ensured that residents can flow in and out of a broad swath of resident activists as their circumstances dictate, though their collective efforts and relationships formed therein, provide a great incentive for them to stay in the community long-term.

GO! Austin/VAMOS! Austin has learned how to be more flexible as new realities and realizations emerge. In more recent years of GAVA, the most important change is the formal incorporation of community organizing into the model and staffing. Organizers—many of whom live in the zip codes of focus—exist both at the geographic level, working with site-based teams, and across sites at the sector level—setting aside the subject matter leads for coordinated school health, early childhood, and physical activity efforts, and leading food retail efforts. Responsible for resident engagement and leadership building within their designated scope, it is these organizers and the Executive Director who provide much of the leadership, outreach, and sense check of what will work. They are the connection between the power of evidence and the reality of lived experience by those most directly impacted.

## Author Contributions

AH, CP, SB, and NR can confirm that they are authors of the mentioned manuscript, and that their authorship consisted of the following: substantial contributions to the conception or design of the work; or the acquisition, analysis, or interpretation of data for the work; and drafting the work or revising it critically for important intellectual content; and final approval of the version to be published; and agreement to be accountable for all aspects of the work in ensuring that questions related to the accuracy or integrity of any part of the work are appropriately investigated and resolved.

## Conflict of Interest Statement

The authors declare that the research was conducted in the absence of any commercial or financial relationships that could be construed as a potential conflict of interest. The reviewer MHF and the handling Editor declared their shared affiliation.
